# Flavonoids Regulate Redox-Responsive Transcription Factors in Glioblastoma and Microglia

**DOI:** 10.3390/cells12242821

**Published:** 2023-12-12

**Authors:** Natali Joma, Issan Zhang, Germanna L. Righetto, Laura McKay, Evan Rizzel Gran, Ashok Kakkar, Dusica Maysinger

**Affiliations:** 1 Department of Pharmacology and Therapeutics, McGill University, 3655 Promenade Sir-William-Osler, Montreal, QC H3G 1Y6, Canada; natali.joma@mail.mcgill.ca (N.J.); issan.zhang@mail.mcgill.ca (I.Z.); germanna.righetto@mail.utoronto.ca (G.L.R.); evan.gran@mail.mcgill.ca (E.R.G.); 2 Structural Genomics Consortium, University of Toronto, 101 College St, Toronto, ON M5G 1L7, Canada; 3 Department of Chemistry, McGill University, 801 Sherbrooke St W, Montreal, QC H3A 0B8, Canada; laura.mckay@mail.mcgill.ca (L.M.); ashok.kakkar@mcgill.ca (A.K.)

**Keywords:** microglia, glioblastoma, tumor microenvironment, reactive oxygen species, oxidative stress, natural polyphenols, fisetin, quercetin, redox-responsive transcription factors

## Abstract

The tumor microenvironment (TME) has emerged as a valuable therapeutic target in glioblastoma (GBM), as it promotes tumorigenesis via an increased production of reactive oxygen species (ROS). Immune cells such as microglia accumulate near the tumor and its hypoxic core, fostering tumor proliferation and angiogenesis. In this study, we explored the therapeutic potential of natural polyphenols with antioxidant and anti-inflammatory properties. Notably, flavonoids, including fisetin and quercetin, can protect non-cancerous cells while eliminating transformed cells (2D cultures and 3D tumoroids). We tested the hypothesis that fisetin and quercetin are modulators of redox-responsive transcription factors, for which subcellular location plays a critical role. To investigate the sites of interaction between natural compounds and stress-responsive transcription factors, we combined molecular docking with experimental methods employing proximity ligation assays. Our findings reveal that fisetin decreased cytosolic acetylated high mobility group box 1 (acHMGB1) and increased transcription factor EB (TFEB) abundance in microglia but not in GBM. Moreover, our results suggest that the most powerful modulator of the Nrf2-KEAP1 complex is fisetin. This finding is in line with molecular modeling and calculated binding properties between fisetin and Nrf2-KEAP1, which indicated more sites of interactions and stronger binding affinities than quercetin.

## 1. Introduction

Glioblastoma multiforme (GBM) is an aggressive brain cancer of glial origin. Its heterogeneity and propensity for treatment resistance (innate or acquired) are significant challenges in the clinics [1,2]. The GBM microenvironment contains a significant proportion of tumor-associated macrophages (TAMs) and microglia (40–50%) [3,4,5]. Microglia are immunological surveyors of the brain, which respond to signals and stressors with a repertoire of neurotrophic factors and inflammatory mediators [6,7,8]. Under maladaptive conditions, microglia can excessively release pro-inflammatory cytokines, damage-associated molecular patterns (DAMPs), and reactive oxygen species (ROS), contributing to neuroinflammation and redox imbalance [6]. The pharmacological modulation of microglia has the potential to reduce injury in neurodegenerative disorders, brain injury, and cancer [1,4,5,9,10,11]. 

One of the key dysregulated factors in GBM is high mobility group box 1 (HMGB1) [6,12]. HMGB1’s activity is influenced by its cellular localization, oxidative state, and post-translational modifications, such as acetylation, methylation, and phosphorylation [13]. HMGB1 mediates inflammatory responses by binding to receptors like RAGE (receptor for advanced glycation end products) and TLRs (Toll-like receptors). Extracellular HMGB1 can activate immune cells, including microglia, and promote tumor invasiveness, resistance, and immunosuppression [14,15]. HMGB1 interacts with various cellular factors, including heat shock protein 72 (HSP72), a chaperone known for mediating resilience against oxidation, inflammation, and other stressors [16,17,18].

Another critical redox-responsive factor in inflammation is transcription factor EB (TFEB), the primary regulator of lysosomes [19]. Under conditions of stress, TFEB translocates to the nucleus, signaling increased lysosomal biogenesis and function. This translocation is mediated by an upstream transcription factor, nuclear factor erythroid-derived-2-like 2 (Nrf2). Under homeostasis, Nrf2 remains bound to Kelch-like ECH-associated protein 1 (KEAP1). However, in response to stress, Nrf2 dissociates from KEAP1, leading to enhanced antioxidant defenses and oxidative stress regulation [20].

Flavonoids, known for their therapeutic activity against a range of diseases, including cancer, cardiovascular disease, and neurodegenerative disorders [21], exhibit varying effects on cells and organs. These differences can be in part attributed to the complexity of human tissues and the diversity of cellular responses, particularly in the brain [22,23,24]. These differences are particularly noticeable when comparing cancer cells with non-transformed cells. Many polyphenols display cytoprotective properties in normal cells and cytotoxic effects in cancer cells (e.g., GBM) [25,26]. These findings prompted the investigation of potential interactions between natural polyphenols (fisetin and quercetin) and several molecular targets, focusing on microglia as sensitive responders in the GBM microenvironment. 

The treatment of microglia and GBM using natural compounds represents an attractive approach to undermine the supportive role of TAMs in the GBM microenvironment by targeting multiple dysregulated pathways in inflammation. However, the interaction between flavonoids with transcription factors implicated in cell functions was not previously explored. Here, we examined the modulatory effects of fisetin and quercetin on major stress-responsive factors (TFEB, Nrf2, KEAP1, HSP72, and HMGB1) in human microglia and GBM cells [27,28,29,30,31]. These agents were selected because of their documented anti-inflammatory properties [21,32,33]. 

The aim of this study was to establish the differential effects of natural polyphenols in human microglia and GBM cells. We first evaluated the toxicity of the polyphenolic compounds and their capacity to modulate (i) reactive oxygen species (ROS) and (ii) the abundance and location of HMGB1 and TFEB. We then investigated protein–protein interactions of Nrf2, KEAP1, HSP72, and HMGB1 in parallel with molecular docking to reveal interactions between the polyphenols and proteins of interest. This study should advance the current understanding of the selected natural compounds as differential modulators of microglia and GBM. 

## 2. Materials and Methods

### 2.1. Cell Culture

The HMC3 human microglia (CRL-3304) and U251N human glioblastoma cells were originally obtained from the American Type Culture Collection. Unless otherwise specified, cells were maintained in Dulbecco’s Modified Eagle Medium (DMEM, 11965084, Thermo Fisher Scientific, Ottawa, ON, Canada) supplemented with 5% (*v*/*v*) fetal bovine serum (Wisent, St-Jean-Baptiste, QC, Canada) and 1% (*v*/*v*) Penicillin–Streptomycin (Thermo Fisher Scientific, Ottawa, ON, Canada) at 37 °C with 5% CO_2_ and 95% relative humidity.

### 2.2. Mitochondrial Metabolic Activity (MTT) 

Cells were seeded at 50,000 cells per well in 24-well plates (Sarstedt, Montreal, QC, Canada) and cultured for 24 h before treatment. Cells were washed twice with phosphate-buffered saline (PBS) before treatment with increasing concentrations of fisetin (5, 15, 25, 50, 100 μM; Cayman Chemical Company, Ann Arbor, MI, USA) or quercetin (5, 15, 25, 50, 100 μM; Cayman Chemical Company, Ann Arbor, MI, USA) in serum-deprived DMEM for 24 h. Cells were then incubated with 0.5 mg/mL MTT (Millipore-Sigma, Oakville, ON, Canada) at 37 °C. The medium was removed, and cells were lysed with dimethylsulfoxide (DMSO, Millipore-Sigma, Oakville, ON, Canada) before colorimetric measurements at 595 nm (Spark 10M, Tecan, Männedorf, Switzerland). 

### 2.3. Lactose Dehydrogenase (LDH) Assay 

Cells were seeded and treated for the MTT assay. At the end of treatment, the medium was collected and centrifuged at 14,000 rpm for 5 min at 4 °C to remove cell debris. The supernatant was then used according to the instructions of the Cytotoxicity Detection Kit (Roche, Mississauga, ON, Canada). In short, medium samples were pipetted in triplicate in a 96-well plate (Sarstedt, Montreal, QC, Canada) and incubated with the kit reagent for 30 min at room temperature in the dark. Colorimetric measurements were made at 492 nm (Spark 10M).

### 2.4. Cell Counting 

Confluent monolayer cell cultures of 70–80% were detached using 0.05% trypsin–EDTA, seeded in 96-well cell culture plates (Sarstedt) at 2500 cells per well, and cultured for 24 h. Cells were treated with increasing concentrations of TMZ (0.001, 0.5, 1, 10, 50, and 100 μM) +/− fisetin (IC50 = 10 μM) for 72 h. Nuclei were labeled with Hoechst 33342 (10 μM, Thermo Fisher Scientific) for 30 min. Cells were imaged using a fluorescence microscope (Leica DMI4000B) with the UV filter at 10× magnification. 

### 2.5. CellROX Assay 

Cells were seeded at 7000 cells per glass coverslip and cultured for 24 h. Cells were washed twice with phosphate-buffered saline before treatment. Cells were treated with fisetin (25 μM) or quercetin (25 μM) with or without buthionine sulfoximine (BSO, 100 μM, Millipore-Sigma, Oakville, ON, Canada) or SARS-CoV-2 spike protein (SMT1-1, 5 μM, National Research Council Canada, Ottawa, ON, Canada). Following treatment, cells were incubated for 30 min at 37 °C with CellROX Deep Red (5 μM, Thermo Fisher Scientific, Burlington, ON, Canada) to detect intracellular oxidative stress. Cell nuclei were labeled with Hoechst 33342 (10 μM, Thermo Fisher Scientific, Burlington, ON, Canada). Cells were rinsed in phenol-free DMEM once and imaged using a fluorescence microscope with the CY5 filter at 20× magnification (Leica DMI 4000B, Richmond Hill, ON, Canada).

### 2.6. Immunocytochemistry 

Cells were seeded at 7000 cells/coverslip on glass coverslips and cultured for 24 h before treatment. Following treatment, cells were fixed in 4% paraformaldehyde (10 min), permeabilized with 0.1% Triton X-100 (*v*/*v*, 10 min, Sigma-Aldrich, St. Louis, MO, USA), blocked in 10% goat serum (*v*/*v*, 1 h, Thermo Fisher Scientific) in PBS, and incubated with primary antibodies overnight at 4 °C, namely rabbit anti-TFEB (1:500, Sigma-Aldrich, SAB4503154-100UG) or rabbit anti-HMGB1 acetyl-Lys12 (1:500, MyBioSource, MBS9404216, San Diego, USA). Cells were washed in PBS three times and incubated with secondary antibodies for 1 h at room temperature: goat anti-rabbit Alexa Fluor 647 (1:500, Thermo Fisher, A21244). Cells were washed with PBS; nuclei and F-actin were labeled with Hoechst 33342 (10 μM, 10 min) and F-actin with 1:400 Alexa Fluor^®^ 488 Phalloidin (Invitrogen, Burlington, ON, Canada), respectively. After three more washings with PBS, coverslips were mounted on microscope slides using Aqua-Poly/Mount (Polysciences, Warrington, PA, USA). Samples were imaged using a fluorescence microscope (Leica DMI4000B, Leica), and intracellular fluorescence was analyzed in ImageJ. The nuclear and/or cytoplasmic fluorescence of TFEB and acetylated HMGB1 for each cell was measured and normalized to the nuclear or cytoplasmic area. The background fluorescence was subtracted. 

### 2.7. GBM Tumoroid Preparation 

U251N tumoroids were prepared using the hanging drop method [34]. Drops of 5000 cells in 20 μL medium were pipetted onto the inner side of the lid of a 100 mm Petri dish (Thermo Fisher Scientific). The lid was quickly flipped 180° to cover the Petri dish filled with 20 mL PBS. Hanging drops were cultured at 37 °C for 48 h to allow tumoroids to form. Tumoroids were then gently scooped into a medium-filled Petri dish coated with 2% agarose and cultured for 48 h. Tumoroids were implanted in agarose gel. The gels were covered with 100 μL DMEM with or without treatment. Tumoroids were imaged using light microscopy immediately after implantation on day 0 and day Tumor size (area) was measured using ImageJ.

### 2.8. Molecular Docking 

The crystal structures of HMGB1 (PDB: 1AAB), HSP72 bound to ADP (PDB ID: 5BN9), and KEAP1 bound to Nrf2 (PDB ID: 3WN7) were selected for docking analysis because of their high resolution and overall high-quality scores among the proteins deposited in the Protein Data Bank for each target. Prior to the docking, Pymol (The PyMOL Molecular Graphics System, Version 1.2r3pre, Schrödinger, and LLC) was used to edit the protein structures and delete any undesired ligands or binding partners. The SwissDock web server was used for molecular docking, and well-characterized binding pockets were defined as regions of interest [35]. In the case of HSP72 and KEAP1, the docking was restricted to the binding region of the compound co-crystallized with the proteins. For the apo HMGB1 structure, previous docking and NMR information of protein binding to glycyrrhizin and salicylic acid was used to define the likely binding region for the natural compounds [36,37,38]. The software UCSF Chimera (version 1.14.0) was used to analyze the docking results and curate the most promising binding modes for each molecule [39]. Binding modes with the most favorable energy (low DG) and making more contact with the protein binding region were chosen for further analysis. The software LigPlot+ (version 2.2.8) was used to investigate the characteristics of protein-ligand binding, such as contacting amino acids and interaction strengths [40]. PyMol software (version 2.4.0) was used to create 3D representations of the results observed on LigPlot+ for the binding modes with a higher number of contacting amino acids and a higher number of hydrogen bonds and hydrophobic contacts for each natural compound analyzed.

### 2.9. Proximity Ligation Assay 

Cells were seeded at 7000 cells per glass coverslip and cultured for 24 h before treatment. At the end of treatment, cells were washed twice with PBS, fixed with 4% paraformaldehyde (10 min, Millipore-Sigma, Oakville, ON, Canada), and then permeabilized with 0.1% Triton X-100 (*v*/*v*), 10 min (Millipore-Sigma, Oakville, ON, Canada). Blocking was performed for 1 h following instructions from the Duolink kit (Thermo Fisher Scientific, Oakville, ON, Canada). Samples were incubated with primary antibodies (rabbit anti-Nrf2, Abcam, Toronto, ON, Canada, 1/500; mouse anti-KEAP1, Proteintech, Rosemont, Illinois, USA, 1/500; rabbit anti-HMGB1, Abcam, Toronto, ON, Canada, 1/500; mouse anti-HSP70, Abcam, Toronto, ON, Canada, 1/500) overnight at 4 °C in a humidified chamber. Samples were then washed and processed for proximity ligation following the manufacturer’s recommendations. Nuclei were labeled for 10 min with Hoechst 33342 (10 μM), and actin was labeled for 20 min with Phalloidin Alexa Fluor 488 (1/400, Thermo Fisher Scientific, Oakville, ON, Canada). Samples were imaged using a fluorescence microscope (Leica DMI 4000B).

### 2.10. Western Blot 

Cells were seeded at 300,000 cells per dish in 35 mm cell culture dishes (Thermo Fisher Scientific, Oakville, ON, Canada) and cultured for 24 h. Cells were treated in serum-deprived conditions for 24 h, after which cells were washed twice with cold PBS on ice. Cells were harvested with cell scrapers and centrifuged at 2500 rpm and 4 °C for 3 min, resuspended in lysis buffer (RIPA) supplemented with complete inhibitors (Roche, Mississauga, ON, Canada) and incubated for 30 min on ice. Cell lysates were centrifuged at 14,000 rpm and 4 °C for 30 min, after which the supernatants were used for SDS-PAGE after protein quantification following instructions from the Pierce BCA assay (Thermo Fisher Scientific, Oakville, ON, Canada). Samples were normalized to 15 μg protein per lane and boiled for 5 min at 95–100 °C in the presence of Laemmli buffer. Samples were loaded onto 10% bis-acrylamide gels and run at 100 V for 30 min and then 120 V for 1 h. Proteins were transferred onto PVDF membranes (Bio-Rad, Saint-Laurent, QC, Canada) at 250 mA for 90 min on ice. Blots were blocked in 5% BSA-TBS-T (Wisent, St-Jean-Baptiste, QC, Canada) for 1 h at room temperature and then incubated with primary antibodies in 5% BSA-TBS-T overnight at 4 °C (mouse anti-KEAP1, Abcam, 1/4000; mouse anti-HSP70, Abcam, 1/4000; rabbit anti-HMGB1, Abcam, 1/4000; mouse anti-beta-tubulin, Cell Signaling, Burlington, ON, Canada). Blots were washed three times for 10 min in TBS-T and then incubated with secondary antibodies in 5% BSA-TBS-T (goat anti-mouse HRP, Bio-Rad, 1/5000; goat anti-rabbit HRP, Bio-Rad, 1/5000, Saint-Laurent, QC, Canada) for 1 h at room temperature. Blots were washed three times in TBS-T for 10 min and then incubated with chemiluminescent substrate (Clarity, Bio-Rad, Saint-Laurent, QC, Canada) before imaging with Imager 600 (Amersham, Oakville, ON, Canada). Band density was analyzed in ImageJ (version 2.14.0).

### 2.11. Statistical Analysis 

Data are presented as mean and standard deviation (SD) (mean ± SD). Statistical significance was determined using a two-way analysis of variance (ANOVA), followed by Tukey’s multiple comparison test. *p*-values less than 0.05 were considered significant. Experiments were repeated independently at least three times. Statistical analysis and graph representations were performed using GraphPad Prism (version 10.0.0). 

## 3. Results

### 3.1. GBM Cells Are More Sensitive to Fisetin and Quercetin Compared to Microglia 

Our study focuses on two well-studied flavonoids, fisetin and quercetin, known for their potential pharmacological relevance. Specifically, these natural compounds have shown remarkable anti-cancer effects in numerous selected in vitro and in vivo systems [41]. The physicochemical properties of fisetin and quercetin are summarized in Table 1. 

**Figure 1:**
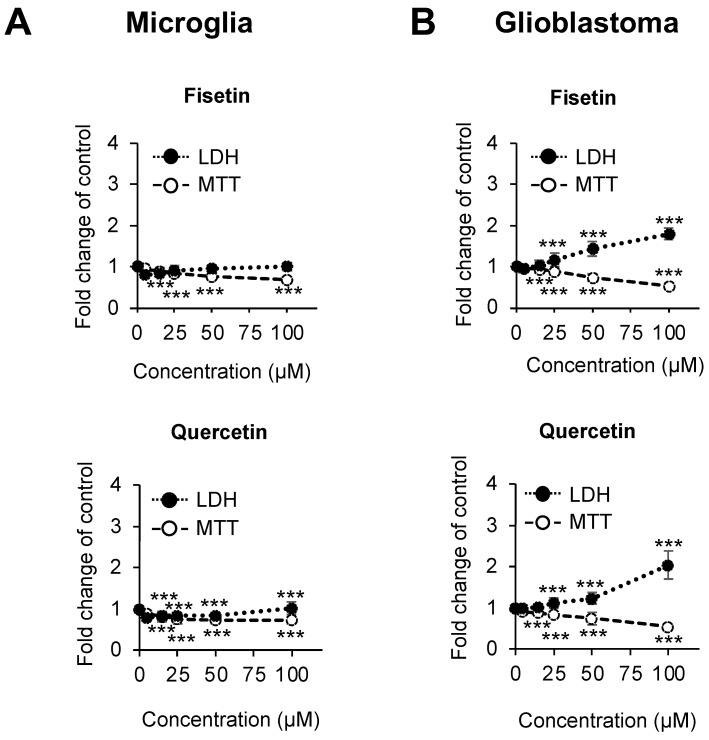
Dose-dependent lactate dehydrogenase (LDH) release and mitochondrial metabolic activity (MTT) in response to fisetin and quercetin. (**A**) Human microglia and (**B**) GBM cells were treated with increasing concentrations of selected compounds (0, 5, 15, 25, 50, 100 μM for fisetin and quercetin) for 24 h in serum-deprived conditions. Culture medium was used to assess LDH release, and cellular mitochondrial metabolic activity was measured using the MTT assay. Shown are the average fold change in LDH or MTT compared to the mean of the untreated control (set to 1) ±SD from at least 3 independent experiments. (*** *p* < 0.001).

We first compared the cytotoxicity of the selected natural compounds on the mitochondrial metabolic activity (MTT) and lactate dehydrogenase (LDH) release of microglia and GBM cells. Cells were cultured with increasing concentrations of fisetin or quercetin (0, 5, 15, 25, 50, 100 μM). These compounds had minimal impact on the viability of microglia (Figure 1A). Fisetin and quercetin reduced GBM cell viability in a dose-dependent manner, indicating differential cytotoxicity in these cell types (Figure 1B). 

### 3.2. Combination of Fisetin and Temozolomide Synergistically Inhibits GBM Survival

**Figure 2:**
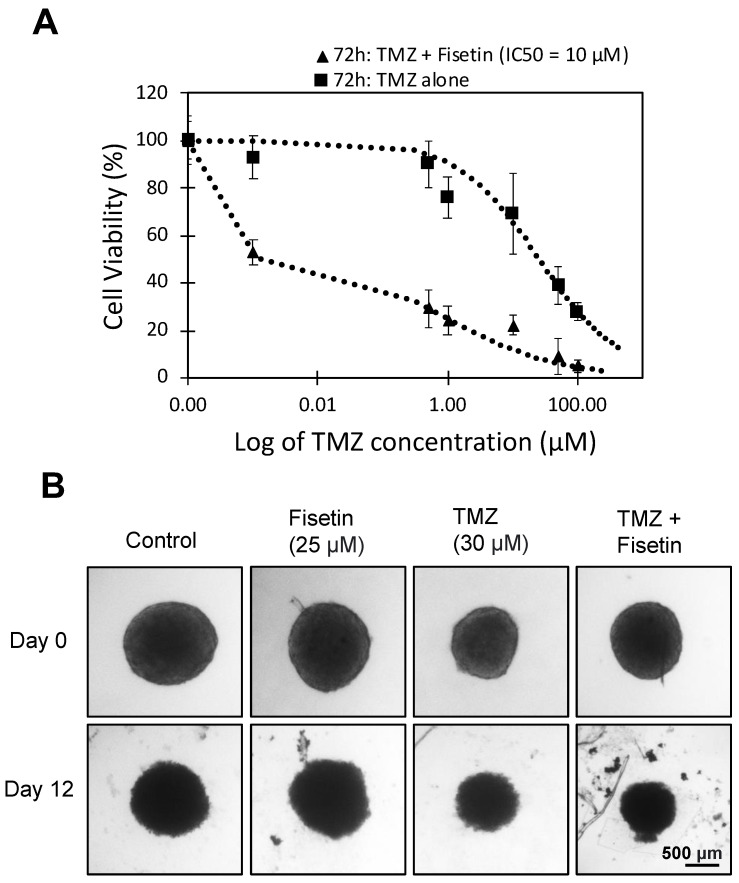
Dose-dependent effect in response to fisetin and TMZ. (**A**) Dose-dependent decrease in cell viability (72 h) with the combination of a fixed concentration of fisetin (10 μM) and increasing concentration of TMZ (0.001, 0.5, 1, 10, 50, and 100 μM). Each point represents the mean from three independent experiments normalized to the untreated control. Cell viability was measured by counting Hoechst 33342-labeled nuclei imaged using a fluorescence microscope. (**B**) Representative micrographs of GBM tumoroids treated with TMZ (30 μM) +/− fisetin (25 μM) on day 0 and day 12. Tumor size (area) was calculated using ImageJ.

While natural compounds may not directly induce cancer cell death, they have the potential to improve the potency of GBM treatment when combined with anti-cancer drugs, such as temozolomide (TMZ). We assessed the effect of combination treatment with fisetin and TMZ by cell counting and MTT assay, followed by calculating the combination index (CI). The combination of fisetin and TMZ at their respective IC50 concentrations proved to be more effective than either compound alone. When 10 μM fisetin was combined with 30 μM TMZ, there was a 70% reduction in GBM cell viability after 72 h (Figure 2A). Notably, the CI value for the fisetin-TMZ combination was 0.00013 (C < 1), indicating a synergistic effect.

Subsequently, we conducted additional testing of these selected compounds on GBM tumoroids, which serve as a more relevant model in vivo. Given that tumoroids are more drug-resistant than monolayer cultures, we used a higher concentration of fisetin (25 μM). In line with results obtained from monolayer cultures, the combination of fisetin and TMZ resulted in a reduction in tumoroid area, whereas treatment with individual compounds did not noticeably impact tumor size (Figure 2B).

### 3.3. Fisetin Reduces Oxidative Stress in GBM and Microglia

**Figure 3:**
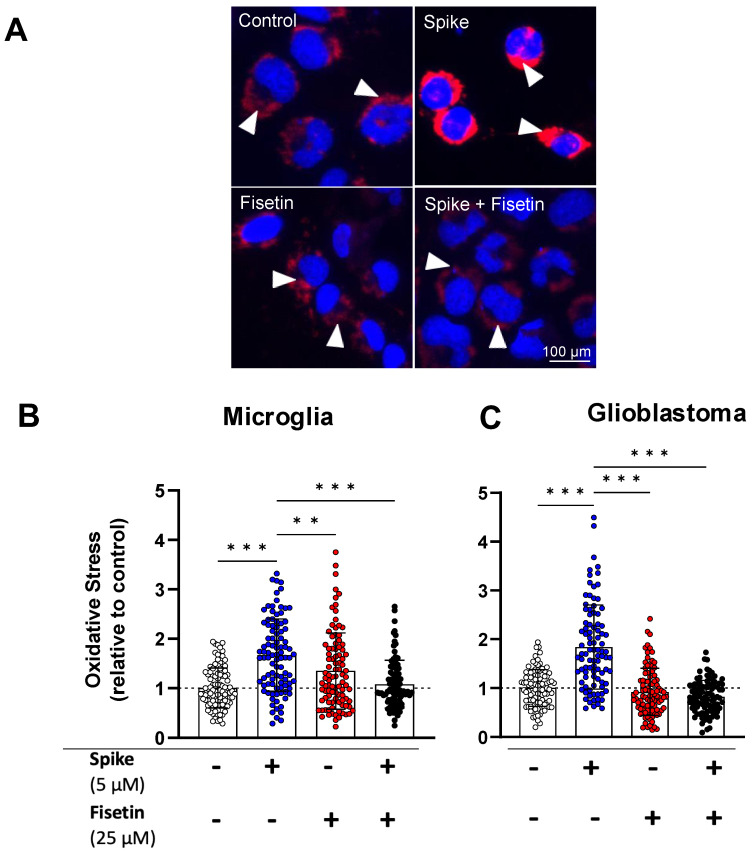
Oxidative stress in human microglia and GBM cells treated with fisetin +/− spike. Microglia and GBM cells were treated with spike (5 μM), fisetin (25 μM), or a combination of spike and fisetin for 24 h in serum-free media. (**A**) Representative fluorescence micrographs of microglia loaded with CellROX Deep Red (red) and imaged using a fluorescence microscope. Nuclei (blue) were labeled with Hoechst 33342. The arrowheads represent intracellular ROS. Scale bar = 100 μm. Shown are the normalized intracellular fluorescence values to the mean of the untreated control (set to 1) for (**B**) microglia and (**C**) GBM. Dotted lines represent the mean of the control group normalized to 1. Statistical analysis was assessed using two-way ANOVA, followed by Tukey’s multiple comparison test. At least 90 cells from three independent experiments were analyzed. Mean ± SD. (** *p* ≤ 0.01, *** *p* ≤ 0.001).

Many natural compounds have been shown to modulate oxidative stress, which is sustained differently in normal and transformed cells. Oxidative stress enhances cancer cell invasiveness and supports glioblastoma stem cell maintenance [42], contributing to treatment resistance and tumor recurrence [43]. Environmental stressors like chemicals, pollutants, and radiation are known triggers for oxidative stress [44]. Previously, we established that microglia could be activated by lipopolysaccharide (LPS), an element of Gram-negative bacterial cell walls [45]. In this study, we investigated the antioxidant effects of fisetin and quercetin in both microglia and GBM cells when subjected to exogenous stressors, namely L-buthionine sulfoximine (BSO) (Figure S1) or SARS-CoV-2 spike protein (SMT1-1) (Figure 3). 

To confirm the effects on ROS status, we detected changes in intracellular ROS using the fluorescent probe CellROX. Fisetin and quercetin proved most efficient at decreasing ROS levels in microglia (Figure S1A) back to control or lower, whereas only fisetin exhibited effectiveness in GBM cells (Figure S1B). These findings align with previous studies using BSO in combination treatment with natural compounds such as curcumin to kill GBM [46].

We found that spike increased ROS production in both microglia (Figure 3A,B) and GBM cells (Figure 3C). Nevertheless, spike-mediated oxidative stress was significantly attenuated after treating microglia and GBM cells with fisetin (Figure 3B,C). These results show that fisetin treatment normalized ROS levels back to control and suppressed spike-induced oxidative stress in both cell types, indicating a lack of cell type-specific effects of fisetin on ROS modulation. 

### 3.4. Fisetin Increases TFEB Abundance in Stressed Microglia 

**Figure 4:**
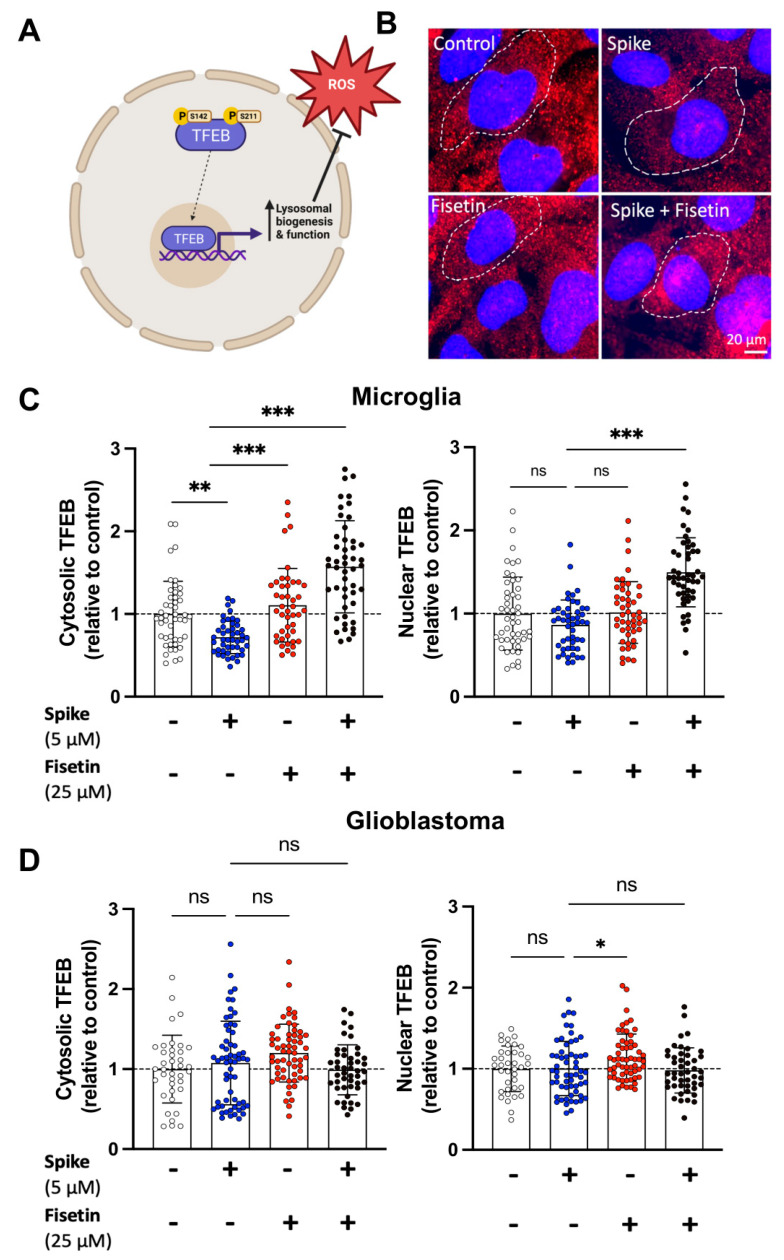
TFEB in human GBM cells and microglia treated with fisetin +/− spike. GBM cells and microglia were treated with spike (5 μM), fisetin (25 μM), or a combination of spike and fisetin for 24 h in serum-free media. (**A**) TFEB undergoes dephosphorylation under conditions of stress, where it is free to translocate to the nucleus and upregulate transcription of lysosomal biogenesis and function. (**B**) Microglia were labeled for TFEB (red) using rabbit anti-TFEB (1:500) and imaged using a fluorescence microscope. Nuclei were labeled with Hoechst 33342 (blue). Scale bar = 20 μm. Shown are the normalized intracellular fluorescence values to the mean of the untreated control (set to 1) in (**C**) human microglia and (**D**) GBM cells. Dotted lines represent the mean of the control group normalized to 1. Statistical analysis was assessed using two-way ANOVA, followed by Tukey’s multiple comparison test. At least 40 cells from three independent experiments were analyzed. Mean ± SD. (ns—nonsignificant, * *p* ≤ 0.05, ** *p* ≤ 0.01, *** *p* ≤ 0.001).

An important redox-sensitive transcription factor that is altered by oxidative stress is transcription factor EB (TFEB). TFEB regulates the expression of genes that are essential for lysosomal biogenesis and enzymatic activities, including the protease cathepsin B. Normally, TFEB is mainly located in the cytosol; however, under stress, TFEB is translocated to the nucleus, where it promotes the expression of multiple target genes involved in autophagy (Figure 4A) [47]. We used immunocytochemistry (ICC) to evaluate the abundance of nuclear and cytosolic TFEB in microglia and GBM cells treated with fisetin, spike, or their combination. We found that fisetin increased the abundance of TFEB in both the nucleus and cytosol of stressed microglia (Figure 4B,C). Notably, the increase in TFEB levels was less pronounced in GBM cells (Figure 4D). 

### 3.5. Fisetin Decreases Cytosolic acHMGB1 Abundance in Microglia, but Not in GBM

**Figure 5:**
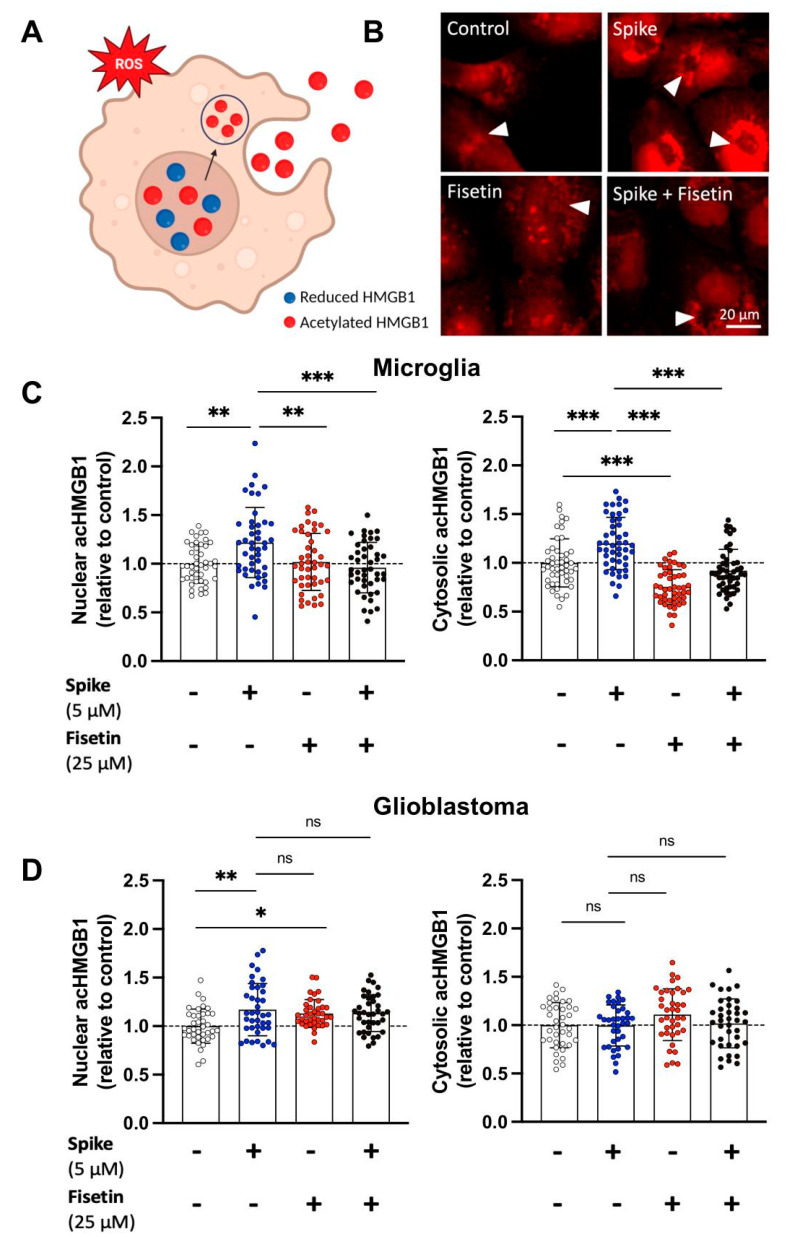
acHMGB1 in human GBM cells and microglia treated with fisetin +/− spike. GBM cells and microglia were treated with spike (5 μM), fisetin (25 μM), or a combination of spike and fisetin for 24 h in serum-free media. (**A**) Under stress, acHMGB1 translocates to the cytoplasm and can be released into the extracellular space as an alarmin. (**B**) Microglia were labeled for acHMGB1 (red) and imaged using a fluorescence microscope. Scale bar = 20 μm. The arrowheads represent cytosolic acHMGB1. Shown are the normalized intracellular fluorescence values to the mean of the untreated control (set to 1) in (**C**) human microglia and (**D**) GBM cells. Dotted lines represent the mean of the control group normalized to 1. Statistical analysis was assessed using two-way ANOVA, followed by Tukey’s multiple comparison test. At least 40 cells from three independent experiments were analyzed. Mean ± SD. (ns—nonsignificant, * *p* ≤ 0.05, ** *p* ≤ 0.01, *** *p* ≤ 0.001).

Another key transcription factor that is redox-responsive is high mobility group box 1 (HMGB1). HMGB1 normally localizes to the nucleus, where it can undergo post-translational modifications, including acetylation (Figure 5A). In response to oxidative stress, it translocates to the cytoplasm and is released in the extracellular space as an alarmin [48]. We used ICC to assess the levels of nuclear and cytosolic acetylated HMGB1 (acHMGB1) in microglia and GBM cells treated with fisetin, spike, or their combination. Fisetin +/− spike significantly reduced cytosolic acHMGB1 in microglia (Figure 5B,C). Interestingly, fisetin did not affect the abundance of cytosolic acHMGB1 in GBM cells (Figure 5D). 

### 3.6. Molecular Docking 

To examine the effects of each natural compound on selected target proteins, we used in silico modeling to calculate binding affinities between proteins and natural compounds in parallel to experimental investigations using proximity ligation assays. The in silico data is depicted graphically (Figures S2 and S3). The calculated binding energies and contact residues are presented in Table 2 and Table 3. 

Cellular ROS is regulated by several factors, including Nrf2, a primary regulator of antioxidant defenses. Under homeostatic conditions, KEAP1 is bound to Nrf2, sequestering the complex in the cytosol. Under oxidative stress, however, the interaction of KEAP1 with Nrf2 is weakened, and thus Nrf2 translocates to the nucleus, activating genes encoding antioxidant, anti-inflammation, and autophagy proteins [49]. HMGB1 is also sensitive to ROS, which causes changes in its intracellular location and post-translational modifications. In turn, HSP72 can serve as a chaperone for proteins modified or damaged by ROS. 

### 3.7. Fisetin and Quercetin Can Bind to KEAP1, HSP72 and HMGB1 

We first investigated if the modulation of Nrf2, HMGB1, and HSP72 could be explained by direct binding of fisetin to the proteins of interest. To restrict the analysis to binding regions with biological relevance, we focused docking on protein regions previously reported as involved in the binding with other compounds or proteins. For HSP72, we focused on the ATP binding pocket, as it is known to bind not only ATP but also other competitive inhibitors [50]. Although there is a lack of structural information on HMGB1 bound to small molecules, biophysical analyses have suggested that its N- and C-terminus regions (box A and B) are involved in the binding of the natural compounds glycyrrhizin and salicylic acid [25,37,38]. Therefore, the box A region was considered in our docking. In the case of KEAP1, the central channel of the protein formed by its beta-propeller folding has been shown to bind different compounds and the protein Nrf2 [51,52,53,54,55].

In silico analyses indicated that fisetin could bind all the proteins of interest with different strengths and predicted binding energies (Figure S2 and Table 2). Fisetin had the highest number of hydrophobic contacts and hydrogen bonds when docked to KEAP1 and HSP72 protein structures, with 10 contacting residues for each protein (Table 2). In addition, both proteins had favorable binding energies to fisetin, with ΔG of −9.1 kcal/mol and −7.9 kcal/mol for KEAP1 and HSP72, respectively. Although fisetin docking to HMGB1 has a similar binding energy (ΔG = −8.1 kcal/mol) compared to other targets, the number of contacts between the compound and protein target is considerably lower (Table 2). This small number of contacts with the compound is related to the overall surface of HMGB1, which has shallow pockets in the box A region (Figure S2F). 

Quercetin, similar to fisetin, is also predicted to bind to all three protein targets (Figure S3). The interaction with KEAP1 presented the most favorable binding energy (−9.1 kcal/mol) and the highest number of contact residues (Table 3). Binding energies were observed to be comparable to that of fisetin (Table 2 and Table 3), and differences in the number of contacting residues were small. Fisetin and quercetin have similar structures (Table 1), featuring a three-ring backbone formed by two benzene rings separated by a pyran ring [56]. The presence of one additional hydroxyl group on quercetin is the major difference between the two molecules.

### 3.8. Modulation of Protein Targets in Microglia and Glioblastoma Cells 

**Figure 6:**
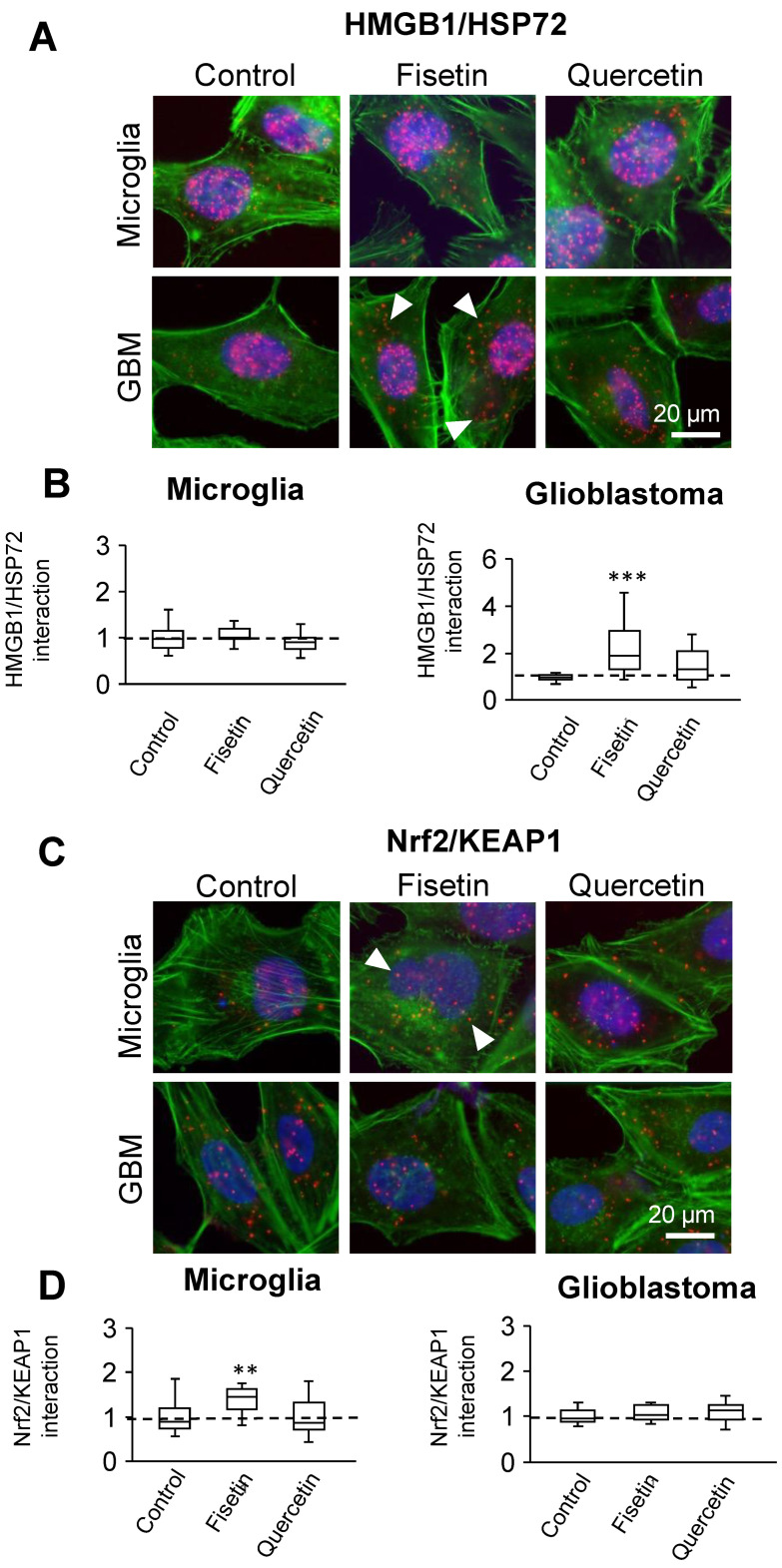
Nrf2-KEAP1 and HMGB1/HSP72 protein–protein interactions detected using proximity ligation assays. Interactions between (**A**,**B**) Nrf2-KEAP1 and (**C**,**D**) HMGB1/HSP72 in microglia and glioblastoma treated with fisetin (25 μM) or quercetin (25 μM) for 24 h in serum-deprived media. Shown are representative fluorescence micrographs with protein interactions (red dots) in cells labeled for actin (green) and nuclei (blue). The arrowheads indicate protein–protein interactions. Shown are the distribution of the number of interactions per cell as fold change in the untreated control (set to 1), with the minimum value, 25th to 75th percentiles, and maximum values indicated. Box blots show the median, 25–75% quartiles, minimum and maximum values. Dotted line represents the mean of the control normalized to 1. At least 60 cells from three independent experiments were analyzed. (** *p* < 0.01; *** *p* < 0.001).

Distinctions between microglia and glioblastoma imply differences in their protein function and interactions. Fisetin and quercetin did not markedly change the protein levels of HMGB1, HSP72, or KEAP1 in microglia and glioblastoma (Figure S4), but did modulate key protein–protein interactions (Figure 6). Results from proximity ligation assays show that fisetin significantly impacts Nrf2-KEAP1 interaction in microglia, whereas it is most effective in enhancing HMGB1-HSP72 interaction in glioblastoma (Figure 6). Overall, these findings support the need for combining in silico modeling with experimental approaches, as each of them contributes to a more complete picture of the biological effects of flavonoids. 

## 4. Discussion

The results obtained from these studies offer insights into the mechanisms of action of fisetin and quercetin within cerebral tumor cells of glioblastoma and microglia, a common central macrophage in the TME. GBM microenvironment plays a pivotal role in tumorigenesis, primarily the generation of ROS. ROS can significantly impact the quantity and quality of non-cancer cells in the vicinity, such as microglia, by modulating redox-responsive transcription factors, including TFEB, Nrf2, HSP72, and HMGB1. The response to treatment observed in microglia and GBM supports the hypothesis that polyphenols exert their differential effects by interacting with redox-responsive factors.

Polyphenols are widely studied bioactive compounds in pharmacology and life sciences and are popularly used in cosmetics, as dietary supplements, and as therapeutics [57,58,59,60,61,62]. A number of studies suggested their potential health benefits in cardiovascular disease, diabetes, and neurodegenerative diseases [32,33,63,64]. They can downregulate inflammation and protect non-cancerous cells against oxidative stress [65,66]. Flavonoids can also inhibit cancer cell proliferation and angiogenesis [67,68,69] with striking cell-type-dependent effects [68,70,71,72,73]. Although their effectiveness in different cell types is well documented, mechanistic data are still sparse. In this study, we compared the effects of fisetin and quercetin in immortalized microglia and transformed GBM cells. GBM cells were more sensitive to the natural compounds, showing pronounced concentration-dependent decreases in mitochondrial metabolic activity, as well as significantly higher LDH release (Figure 1).

Fisetin, a commonly found dietary flavonoid, has recently gained attention for its antioxidant properties and its potential to prevent a broad range of life-threatening diseases [74]. Our study shows that fisetin significantly decreases oxidative stress in both non-neoplastic (microglia) and neoplastic (GBM) cells (Figure 3). Similar to its analog quercetin, fisetin influences numerous biological processes that may contribute to its senolytic effects [75]. For example, due to its hydrophobic nature, fisetin can penetrate and accumulate within cell membranes, where it exerts antioxidant and anti-inflammatory effects [76]. Additionally, fisetin induces apoptotic effects in senescent cells by suppressing Bcl-2 family members and other components of the senescence-associated secretory phenotype (SASP) network [77]. Importantly, fisetin has shown even greater senotherapeutic activity than quercetin in both animal and human tissues [76]. Currently, fisetin is the subject of several clinical trials for various cancers and age-related diseases, including osteoarthritis, coronavirus infections, frail elderly syndrome, and chronic kidney diseases [78]. As a result, the clinical potential of fisetin, in terms of its safety, tolerability, and efficacy, could potentially be harnessed in medicine.

Many natural compounds possess the ability to target multiple molecules, making them valuable candidates for cancer treatment. However, the precise molecular interactions between cellular proteins and neuroprotective polyphenols remain poorly characterized. Our in silico analysis, focusing on KEAP1, HSP72, and HMGB1, suggests that fisetin and quercetin can bind to biologically relevant binding pockets within these proteins. Among these interactions, KEAP1 demonstrates the most favorable binding energy and the highest number of contacts with fisetin and quercetin (Table 2 and Table 3). This observation aligns with the structural characteristics of KEAP1, known for its large central channel typical of WD-repeat proteins [79]. Several co-crystal structures of KEAP1 have implied interactions within its central channel, involving Nrf2 and various small molecules [51,52,53,80,81,82,83]. 

Nrf2 plays a pivotal role in the production of endogenous antioxidants in response to oxidative stress [84,85]. In normal physiological conditions, Nrf2 interacts with the KEAP1 protein in the Kelch domain of KEAP1 and undergoes cytosolic degradation [86]. When mild to moderate oxidative stress occurs, the Nrf2-KEAP1 complex dissociates, allowing Nrf2 to translocate to the nucleus. This stimulates the upregulation of antioxidant-responsive genes such as HO-1 and NQO1, thereby enhancing the production and release of endogenous antioxidants like GSH, SOD, and catalase to mitigate oxidative stress [86,87]. Flavonoids have been reported to interfere with Nrf2-KEAP1 protein–protein interactions in the cytosol, preventing the spontaneous degradation of Nrf2. These flavonoids competitively bind with the KEAP1 protein at the Nrf2 binding site, resulting in the translocation of Nrf2 to the nucleus. This activation of Nrf2 subsequently triggers the upregulation of antioxidant genes like GSH, SOD, and catalase [88]. Increased Nrf2-KEAP1 interaction following fisetin treatment is thus in line with lower intracellular ROS (Figure 3 and Figure 6). 

Fisetin and quercetin also displayed favorable binding energies with HMGB1, although the number of contacts is lower than for KEAP1 (Table 2 and Table 3). HMGB1 is primarily found in the nucleus, but upon stress, it is translocated to the cytosol, where it can be post-translationally modified (acetylated, oxidized, and methylated) [89,90,91,92,93]. HMGB1 can be secreted to the extracellular space via active secretion or passive release by necrotic cells [94,95,96,97,98]. Once in the extracellular environment, it can act as a paracrine factor and interact with receptors, particularly RAGE, thereby activating key signaling pathways that regulate cell growth, differentiation, motility, and death [97,99]. The interaction between HMGB1 and RAGE has been suggested to promote the proliferation and invasion of various tumor cells [99]. Interestingly, we found that fisetin does not affect acHMGB1 abundance and translocation in GBM. On the other hand, fisetin reduces cytosolic acHMGB1 levels in microglia but does not affect nuclear acHMGB1 abundance (Figure 5). This suggests that fisetin modulates the release of acHMGB1 in immune cells and, in turn, reduces the exacerbated inflammation in the TME. Bassi et al. showed that glioma cells contain HMGB1 predominantly in the nucleus and cannot secrete it constitutively or upon stimulation. However, necrotic glioma cells can release HMGB1 after it has translocated from the nucleus to the cytosol [100]. These findings suggest that HMGB1 is acting as an autocrine factor that promotes the growth and migration of tumor cells.

All compounds had comparable binding energies with HSP72, whereas fisetin was predicted to have the greatest number of contacting residues. Fisetin fits deeply into the HSP72 binding pocket, contacting at least three residues at the bottom (Figure S2E). Ser275 was shown as essential for HSP72 to bind ATP and other compounds via hydrogen bonds [101]. HSP72 and its family respond to cellular stressors in both normal and transformed cells [102] and can interact with other stress-responsive proteins, including HMGB1 [103]. HMGB1-HSP72 modulation via fisetin in GBM (Figure 6) is relevant for therapeutic approaches, as HSP70 proteins contribute to drug resistance in many cancers [104,105,106,107]. 

Despite the promising results of flavonoids against various cancer types, their application in cancer treatment is hindered by issues such as low solubility, poor absorption, and rapid metabolism [108]. To overcome these limitations, nanocarriers have been developed to enhance the bioavailability of flavonoids [88]. Both in vitro and in vivo studies have shown potential anticancer activity of flavonoid nanoparticles against A549 lung cancer cells, B16F10 melanoma cells, MCF-7 breast cancer cells, HepG2 liver cancer cells, and CT26 colorectal cancer cells [88]. Various types of flavonoid nanocarriers are currently employed in cancer therapy, including polymeric nanoparticles [109], nanocapsules [110], metallic nanoparticles such as gold [111], and solid lipid nanocarriers [112]. We propose incorporating fisetin into polymeric soft nanoparticles to improve its solubility, stability, and sustained delivery, all while retaining its biological activity (Figure S5). 

Our docking and biological studies aimed at relating potential interactions between fisetin and quercetin with modulations of the protein targets in human cells. The differential effects of the natural compounds depend on the cell type, target protein, and their location, which is often determined by interacting partners and post-translational modifications. Our studies introduce a complementary approach to the development of new therapeutics for GBM, leveraging the simultaneous targeting of multiple intracellular proteins (e.g., transcription factors) whose effects depend on their location and interaction partners. Such an approach could result in more favorable patient outcomes compared to standard therapeutic interventions. 

## 5. Conclusions

Current therapeutic approaches with polyphenols in different pathologies, including GBM, are limited and mainly target the tumor itself. We support the proposed approach that altering the cells in the tumor microenvironment is essential and can contribute to improved patient outcomes. Taken together, the presented studies show differential effects of fisetin and quercetin in microglia and glioblastoma cells. Molecular modeling combined with experimental data suggests that combination with other natural compounds (e.g., docosahexaenoic acid) [113] is warranted and that alternative therapeutic interventions could be developed aiming at differential disruptions of protein–protein interactions in glioblastoma itself and its surrounding microenvironment, comprising microglia, peripheral macrophages, astrocytes, vasculature, etc. In addition to binding energy and contact residues, the affinity and specificity of these interactions need to be further investigated. Future studies using biophysical and structural biology methods will be fundamental to advance our understanding of the proteins-compounds interactions described in this study. Nonetheless, this study suggests direct binding of fisetin and quercetin to the binding pockets of KEAP1, HSP72, and HMGB1. We suggest that experimental approaches, particularly with human organoids consisting of several cell types—transformed and non-transformed—would offer acceptable testing paradigms for future therapeutics. Such experimental models using human cells combined with single-cell transcriptomics and single-nucleus transcriptomics could offer new paths to uncover better treatments for GBM [114]. 

## Figures and Tables

**Table 1:** Physicochemical properties of natural products investigated.

	Molecular Mass (g mol^−1^)	Water Solubility (mg mL^−1^)	Topological Polar Surface Area (Å^2^)	Number of H-Bond Donors	Number of H-Bond Acceptors	λ_max_ in Water (nm)
Fisetin (C_15_H_10_O_6_)	286.24	0.01	107	4	6	360
Quercetin (C_15_H_10_O_7_)	302.23	0.06	127	5	7	557

**Table 2:** Parameters of fisetin binding to target proteins.

Target	ΔG (Kcal mol^−1^)	Hydrophobic Contacts	Hydrogen Bonds	Number of Contacting Residues	Residues in Common with Known Ligand
KEAP 1	−9.1	5	5	10	9
HSP72	−7.9	9	1	10	9
HMGB1	−8.1	3	1	4	2

**Table 3:** Parameters of quercetin binding to target proteins.

Target	ΔG (Kcal mol^−1^)	Hydrophobic Contacts	Hydrogen Bonds	Number of Contacting Residues	Residues in Common with Known Ligand
KEAP 1	−9.1	8	7	13	10
HSP72	−7.2	5	2	7	5
HMGB1	−6.2	6	1	7	3

## Data Availability

Data are contained within the article.

## Supplementary Materials

The following supporting information can be downloaded at https://www.mdpi.com/2073-4409/12/24/2821/s1, Figure S1: Modulation of intracellular oxidative stress induced by buthionine sulfoximine (BSO) using selected natural compounds; Figure S2: Docking analyses of fisetin binding to target proteins; Figure S3: Docking analyses of quercetin binding to target proteins; Figure S4: Protein levels of KEAP1, HSP72 and HMGB1 in human microglia and glioblastoma cells; Figure S5: Physical properties of fisetin-loaded self-assembly.
