# Supplementary material for: Flavonoids Regulate Redox-Responsive Transcription Factors in Glioblastoma and Microglia
Source: Cells. 2023 Dec 12;12(24):2821. doi: 10.3390/cells12242821 (PMC10871111; doi:10.3390/cells12242821)
Supplement: Supplementary file 1 [file cells-12-02821-s001.zip › cells-2725838-supplementary.pdf]

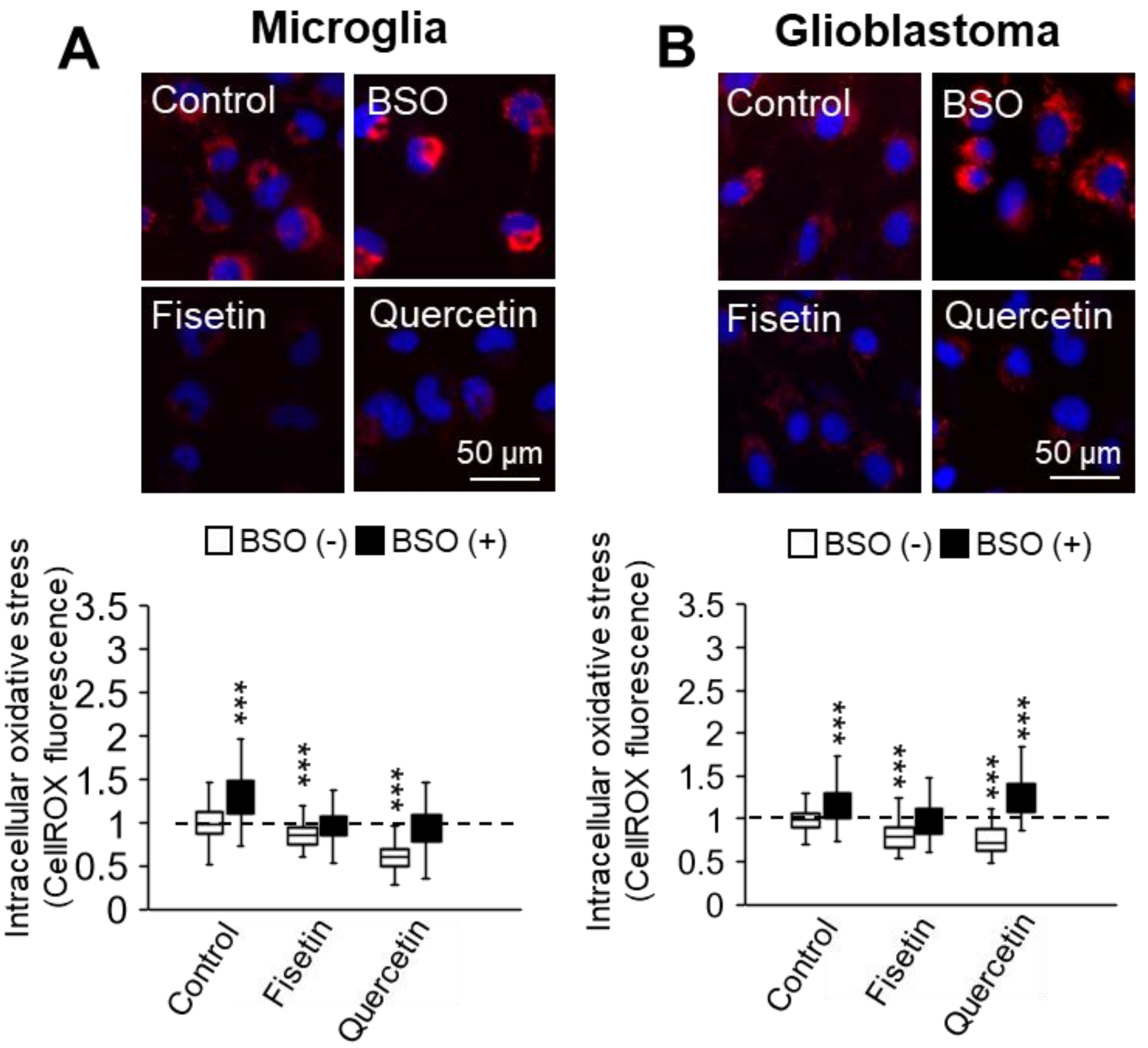

Figure S1. Modulation of intracellular oxidative stress induced by buthionine sulfoximine (BSO) using selected natural compounds. (A) Human microglia and (B) glioblastoma cells were treated with fisetin (25 μM) or quercetin (25 μM) in the presence or absence of the glutathione inhibitor BSO for 24 h in serum-deprived media. Cells were then loaded with CellROX Deep Red and imaged live using a fluorescence microscope. Nuclei (blue) were labeled with Hoechst 33342. Intracellular fluorescence was quantified in ImageJ. Shown are representative fluorescence micrographs and the distribution of intracellular oxidative stress level per cell as fold change of the untreated control (set to 1), with the minimum value, 25th to 75th percentiles, and maximum values indicated. At least 180 cells from three independent experiments were analyzed. (***$p<0.001$)

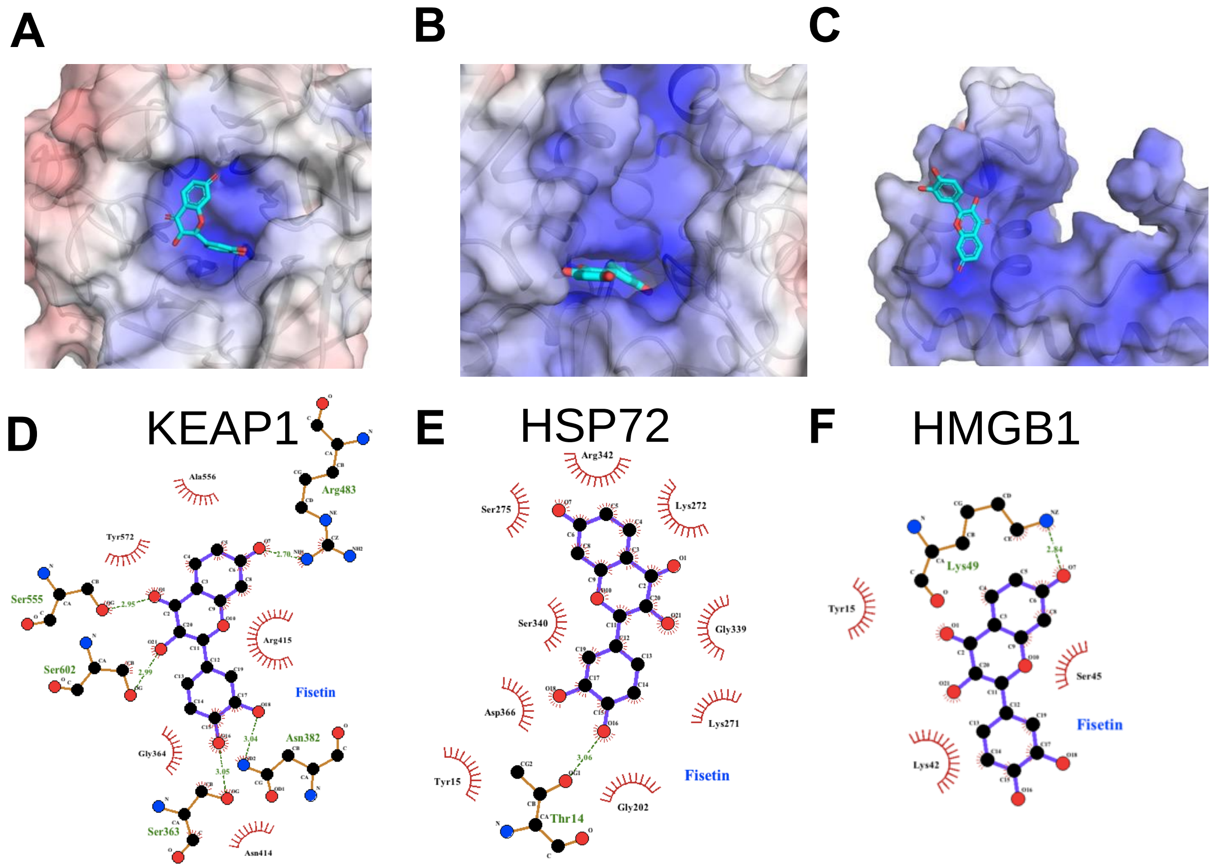

Figure S2. Docking analyses of fisetin binding to target proteins. (A-C) 3D representation of fisetin binding to the protein structures of KEAP1 (PDB ID: 3WN7), HSP72 (PDB ID: 5BN9), and HMGB1 (PDB ID: 1AAB), respectively. Proteins surfaces are colored based on atoms electrostatic potential, shown in a scale of of −10 (red) kT/e to 10 (blue) kT/e. Fisetin is represented as stick and colored in cyan. A cartoon representation of each protein, overlayed with their surface representation, is shown in dark grey. (D-F) 2D representation of the contacting residues and interaction strength of fisetin (purple) interaction with KEAP1, HSP72, and HMGB1, respectively. Hydrogen bonds are represented as green dashed lines and connect the ligand to the respective contacting protein residue (orange). Protein residues performing hydrophobic contacts are displayed as red arcs. The crystal structure of KEAP1 (PDB ID: 3WN7), HSP72 (PDB ID: 5BN9), and HMGB1 (PDB ID: 1AAB) were used for the binding analysis using LigPlot+.

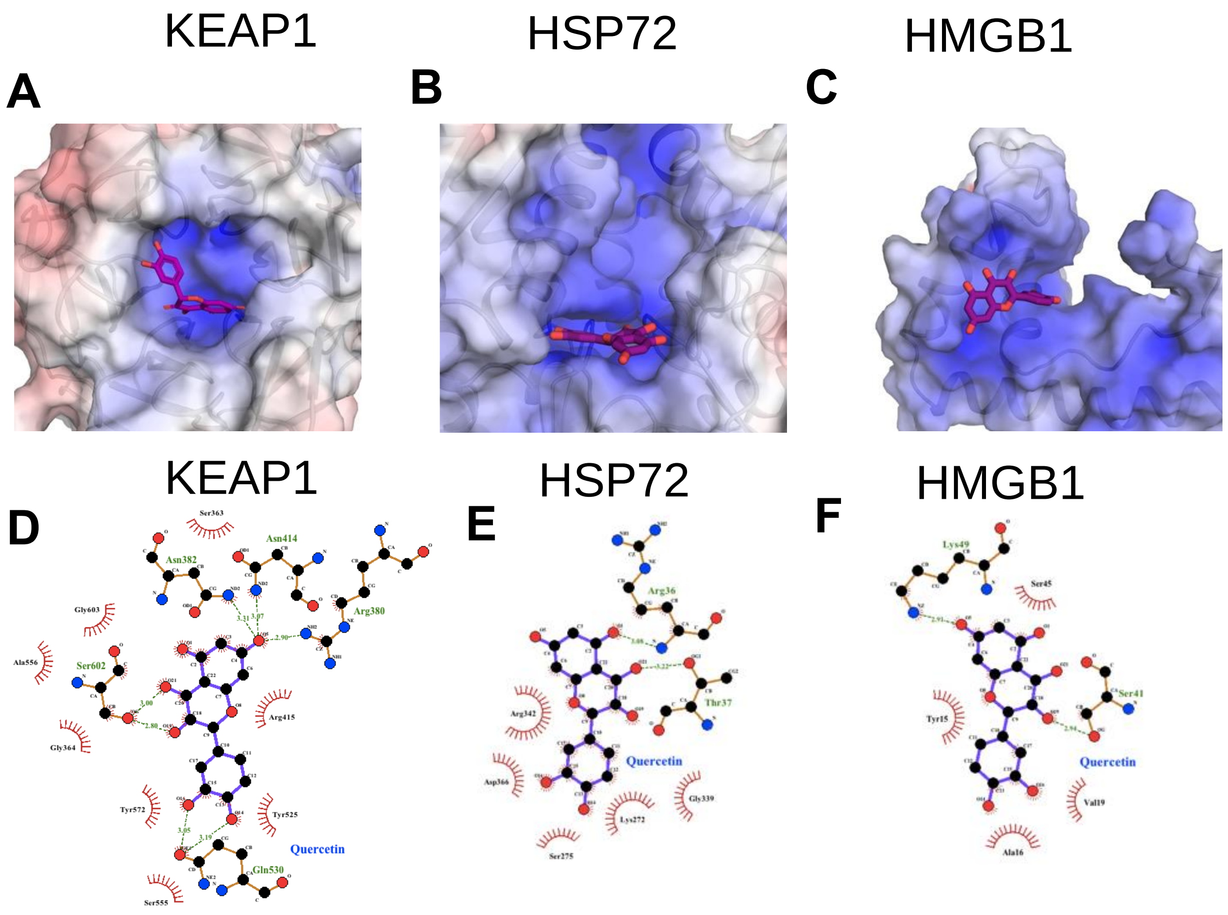

Figure S3. Docking analyses of quercetin binding to target proteins. (A-C) 3D representation of quercetin binding to the protein structures of KEAP1 (PDB ID: 3WN7), HSP72 (PDB ID: 5BN9), and HMGB1 (PDB ID: 1AAB), respectively. Proteins surfaces are colored based on atoms electrostatic potential, shown in a scale of of −10 (red) kT/e to 10 (blue) kT/e. Quercetin is represented as stick and colored in purple. A cartoon representation of each protein, overlayed with their surface representation, is shown in dark grey. (D-F) 2D representation of the contacting residues and interaction strength of quercetin (purple) interaction with KEAP1, HSP72, and HMGB1, respectively. Hydrogen bonds are represented as green dashed lines and connect the ligand to the respective contacting protein residue (orange). Protein residues performing hydrophobic contacts are displayed as red arcs. The crystal structure of KEAP1 (PDB ID: 3WN7), HSP72 (PDB ID: 5BN9), and HMGB1 (PDB ID: 1AAB) were used for the binding analysis using LigPlot+.

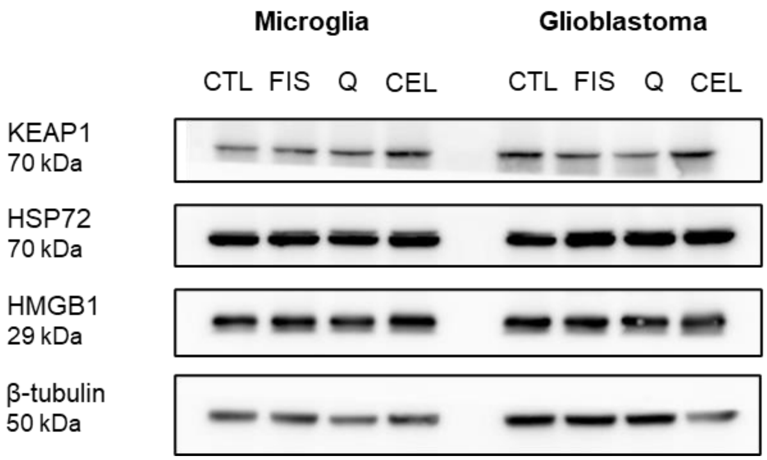

Figure S4. Protein levels of KEAP1, HSP72 and HMGB1 in human microglia and glioblastoma cells. Cells were treated with fisetin (FIS, 25 μM), quercetin (Q, 25 μM) or celastrol (CEL, 0.25 μM) for 24h in serum-deprived media. β-tubulin served as loading control. Shown are representative Western blots from three independent experiments.

A

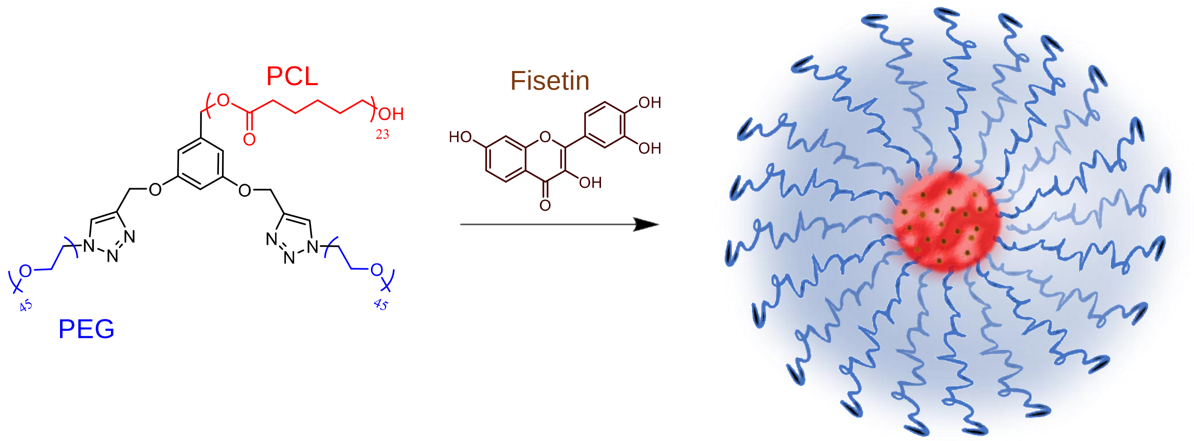

B

Fisetin Loaded Micelles					
Drug: Polymer Feed Ratio	Diameter [c]	Đ [d]	Zeta Potential	EE	LC
1:10	29.2 ± 1.6 nm	0.174 ± 0.015	-6.3 mV	85 %	7.8 %

Figure S5. Fisetin-loaded nanoparticles. (A) Fisetin loading in nanoformulation based on a miktoarm star polymer with AB_2 composition (A = polycaprolactone and B = polyethylene glycol). **(B)** Characterization of fisetin-loaded micelles. EE= encapsulation efficiency; LC = loading capacity.
